# A Rare Case of Imported Cutaneous Leishmaniasis Caused by *Leishmania infantum* in the Republic of Korea, 2021

**DOI:** 10.3390/tropicalmed8040223

**Published:** 2023-04-12

**Authors:** Hyun Jung Kim, Eun Jin Kim, Jee Woong Choi, You Chan Kim, Hee-Il Lee, Hyun-Il Shin

**Affiliations:** 1 Division of Vectors and Parasitic Diseases, Korea Disease Control and Prevention Agency, Cheongju 28159, Republic of Korea; kimhj0324@korea.kr (H.J.K.); isak@korea.kr (H.-I.L.); 2 Department of Medical Science and Infectious Biology, College of Medicine, Chungnam National University, Daejeon 35015, Republic of Korea; 3 Department of Infectious Diseases, Ajou University Hospital, Suwon 16499, Republic of Korea; stone0128@ajou.ac.kr; 4 Department of Dermatology, Ajou University Hospital, Suwon 16499, Republic of Korea; dermaboy@gmail.com (J.W.C.); maychan@ajou.ac.kr (Y.C.K.)

**Keywords:** *Leishmania infantum*, cutaneous leishmaniasis, imported leishmaniasis

## Abstract

Leishmaniasis is a neglected tropical disease and an infectious disease transmitted by sandflies that occurs worldwide. In the absence of physicians seeking to identify the causes of disease in non-endemic areas, appropriate diagnoses cannot be made, thereby hampering effective treatment. In this report, we examined a nodular lesion on a patient’s chin by performing a biopsy and molecular analysis. The biopsy finding led to the identification of a *Leishmania* amastigote. On the basis of PCR analysis of the internal transcribed spacer 1 gene and 5.8 S ribosomal RNA with a subsequent BLAST search, we identified the causal organism as *Leishmania infantum*. The patient, who had visited Spain from 1 July to 31 August 2018, was accordingly diagnosed with cutaneous leishmaniasis and was administered liposomal amphotericin B, which successfully treated the skin lesion. Travel history plays an important role in the diagnosis of leishmaniasis, and physicians should bear in mind that travelers can also introduce diseases and pathogens to non-endemic areas. Identification of *Leishmania* at the species level will increase the efficacy of treatment.

## 1. Introduction

Leishmaniasis is a zoonotic protozoan parasitic disease transmitted by sandflies infected with species of *Leishmania*. According to the World Health Organization, the incidence of leishmaniasis has increased globally over recent decades. Leishmaniasis is endemic in 200 countries in Asia, Africa, America, and the Mediterranean region [1].

Leishmaniasis is classified into three types according to its clinical manifestations, namely, cutaneous leishmaniasis (CL), mucocutaneous leishmaniasis (MCL), and visceral leishmaniasis (VL). Among these, CL is the most common type and is known as Aleppo boil or Baghdad boil or Jericho button [2], with over 600,000 cases reported annually worldwide. It is caused by a range of Leishmania subgenera, including *Leishmania major*, *Leishmania tropica*, *Leishmania aethiopica*, *Leishmania mexicana*, *Leishmania amazonensis*, and *Leishmania braziliensis*. VL, also referred to as post-kala-azar dermal leishmaniasis (PKDL) or black fever or dum-dum fever [2], is the most serious form of the disease, caused by *L. donovani* and *L. infantum*. Although generally considered a causative agent of VL, *L. infantum* can also cause skin and mucosal leishmaniasis [3]. Of the 200 countries in which leishmaniasis is endemic, CL and VL are considered endemic in 89 and 79 countries, respectively, with 71 countries being endemic for both CL and VL [1]. Recently, CL caused by *L. infantum* has been reported in several countries in the Mediterranean region, including France, Italy, Portugal, and Malta. The clinical form of CL, as caused by *L. infantum*, is a single painless papule that progresses to an ulcero-necrotic nodular lesion in a few weeks or months [4,5,6].

To the best of our knowledge, no *Leishmania*-transmitting vector (belonging to the genera *Phlebotomus* and *Lutzomyia* from the Old and New World, respectively) has been reported in the Republic of Korea, in which leishmaniasis is currently non-endemic. However, to date, since the first case of imported visceral leishmaniasis in the Republic of Korea in 1952, a total of 25 imported cases have been reported (20 and 5 cases of CL and VL, respectively). These cases were reported to be associated with travel for professional reasons in areas endemic for *Leishmania*, with patients returning from China, Argentina, and Saudi Arabia [7,8,9,10,11,12,13,14]. The most recent case of imported leishmaniasis was a case of New World CL, in which the infected individual had been bitten by an insect whist travelling in Brazil in 2013 [15]. There are, however, increasing concerns regarding the importation of leishmaniasis from endemic countries to the Republic of Korea. Here, we present a rare case of imported CL via a patient who had traveled to Spain in the Republic of Korea. The infection was confirmed by microscopic examination and molecular analysis. This is the first reported case of imported CL caused by *L. infantum* in the Republic of Korea.

## 2. Case Presentation

**Figure 1:**
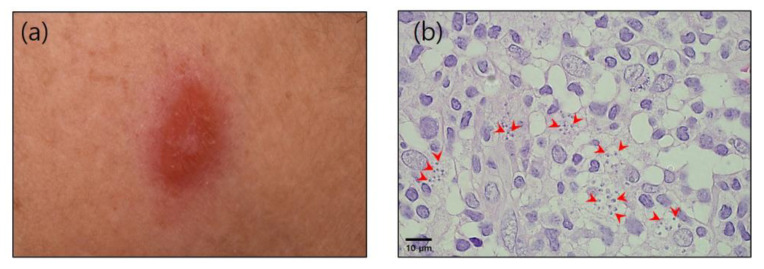
(**a**) Cutaneous nodular lesion caused by *Leishmania* infection on the left side of the patient’s chin (24 May 2021). (**b**) Microscopic appearance of skin lesions with an amastigote form of *Leishmania* (red arrowheads) (hematoxylin and eosin staining; 1000× magnification).

A 78-year-old female patient presented with a 6-month history of a nodular lesion on the left side of her chin. She had a history of hypertension and had traveled to Spain in 2018. She had been living in Gyeonggi in the Republic of Korea, although for 2 months (1 July to 31 August 2018) she had visited her daughter’s house in Tarragona, Spain, an endemic area for leishmaniasis. During her time in Spain, she did not engage in outdoor activities, such as climbing, farming, or camping, and did not recall being bitten by any insects. Moreover, she had no contact with wild animals and did not keep any pets. In January 2021, on examination, a painful and erythematous nodule (10 mm) was detected on the left side of her chin (Figure 1a). She had no fever, and a clinical laboratory examination revealed no abnormalities.

A skin biopsy revealed numerous intracellular cocci-like microorganisms, which led to the diagnosis of chronic granulomatous inflammation. Results obtained from acid-fast bacilli staining, fungal staining, and a polymerase chain reaction (PCR) for tuberculosis were all negative. Although she was treated with different antibiotics and itraconazole 200 mg, there were no improvements in the skin lesion. A further skin biopsy was therefore performed 4 months after the first, which yielded the same findings. Eventually, the possibility of leishmaniasis was considered, based on the fact that the type of microorganism detected was thought to likely be a *Leishmania* amastigote (Figure 1b). The patient was accordingly referred to the Department of Infectious Diseases at the Ajou University School of Medicine.

**Figure 2:**
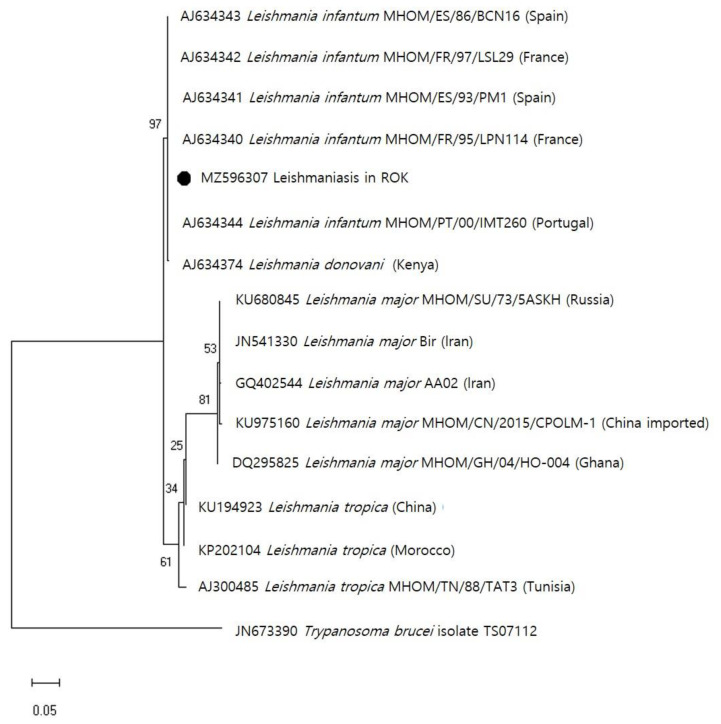
Maximum-likelihood phylogenetic tree of internal transcribed spacer 1 and 5.8 S ribosomal RNA sequences of *Leishmania*. The *Leishmania infantum* sequence identified in this study is indicated by a solid circle (●, GenBank accession no. MZ596307). The scale bar indicates 0.05 nucleotide substitutions per site.

To identify the *Leishmania* species, skin biopsy samples were collected from the patient at Ajou University Medical Center, and microscopic examinations and genetic analyses were performed at the Korea Disease Control and Prevention Agency (KDCA). DNA was extracted from the patient’s skin lesion using a DNeasy Blood and Tissue Kit (Qiagen GmbH, Hilden, Germany) and the *Leishmania* internal transcribed spacer 1 and 5.8 S ribosomal RNA genes were detected based on PCR analysis [16,17]. For species identification, a phylogenetic analysis was performed using the maximum likelihood method to identify the best tree. Analysis was conducted using MEGA software (Pennsylvania State University, State College, PA, USA) version 6, and bootstrap scores were calculated for 1000 replicates. The obtained sequence (314 bp; GenBank accession No. MZ596307) was identified based on a BLAST search. The sequence clustered with *L. infantum* (GenBank accession number: AJ634340-AJ634344) with 100% alignment. These clusters contained *L. infantum* that have been isolated from patients in different regions (the sequences of which are available in GenBank), including Spain (MHOM/ES/93/PM1; AJ634341, MHOM/ES/86/BCN16; AJ634343), France (MHOM/FR/95/LPN114; AJ634340, MHOM/FR/97/LSL29; AJ634342), and Portugal (MHOM/PT/00/IMT260; AJ634344) (Figure 2). Having been diagnosed with *L. infantum*-caused CL, the patient underwent surgical excision and was treated with liposomal amphotericin B at a dose of 3 mg/kg for 15 days. The patient experienced no other side-effects during treatment. The lesion was treated, and no recurrence has been reported.

## 3. Discussion

Human leishmaniasis, which is caused by more than 20 species of *Leishmania*, is classified as a neglected tropical disease, although it continues to be a major health problem in endemic regions.

Leishmaniasis is distinguished based on geographical distribution as Old World (southern Europe, Mediterranean basin, Middle East, Asia, and Africa) or New World (Latin America). It is generally transmitted by the bite of female *Phlebotomus* (Old World) and *Lutzomyia* (New World) sandflies. Although the distribution of sandflies tends to be confined to subtropical and tropical regions between 50° N and 40° S [18,19], leishmaniasis has spread steadily to non-endemic regions in response to climatic and environmental changes, which are promoting the spread of *Leishmania*-transmitting vectors and reservoirs [20,21,22]. In Portugal, for example, the seroprevalence of canine leishmaniasis is 20% higher than that previously reported in endemic foci [23]. In Italy, a comparison with historical data revealed that both the canine reservoir and sandflies have increased and expanded northward [24]. Moreover, sandflies have been detected in Spain at higher altitudes than previously reported [25].

To date, however, there is no evidence to indicate the occurrence of leishmaniasis vectors in the Republic of Korea, thereby limiting the risk of introduction and autochthonous transmission. Nevertheless, although the probability of *Leishmania* spreading to the Republic of Korea is low, notification of imported cases remains necessary, given the rapid growth in international travel. There is currently no vaccine available for leishmaniasis, and, thus, travelers to leishmaniasis endemic areas should be aware that protection from sandfly bites and avoiding outdoor activities when sandflies are most active (from dusk to dawn) are the only practical preventive measures.

Owing to the increasing frequency and intensity of travel and transport over the past century, travelers are generally at a higher risk of infection with *Leishmania* than other individuals [26]. Imported leishmaniasis is typically associated with travelers and migrants who have returned from regions endemic for this disease [27]. According to the recent literature, more than 10,000 cases of leishmaniasis were reported in non-endemic areas between 2000 and 2021, reflecting the increase in global tourism and human migration [28]. According to a recent WHO report, 979 cases (880 of CL and 99 of VL) of imported leishmaniasis were recorded worldwide in 2020 [1]. One study has shown that leishmaniasis is one of the most frequently diagnosed dermatological diseases among travelers, with 3.3% of 4,594 returning travelers contracting travel-related leishmaniasis [29]. Recently, several cases of travel-related leishmaniasis have been reported in other countries. Leishmaniasis surveillance data have revealed that 77% (799/1044) of the leishmaniasis cases recorded in Europe between 2014 and 2019 were travel related [30]. Similarly, 55% of imported VL cases in the UK were found to be related to tourism [31]. Eighty-nine cases of travel-related VL and CL reported in France were established to have originated in America and Africa [32], whereas in Japan, a *Leishmania* non-endemic area, an imported case of CL caused by *L. tropica* was reported to have originated from Pakistan [33].

Microscopic examination is the most widely used method for parasitological diagnosis. Upon Giemsa staining, *Leishmania* amastigotes are observed within macrophages. They appear round in shape, with a diameter of 2 to 4 µm, and have a blue cytoplasm, red nucleus, and purple-pink-stained kinetoplasts [34]. The amastigote stage is the only *Leishmania* stage detected in human hosts. The observation of *Leishmania* amastigotes in stained smears of tissue, such as skin, spleen and lymph node, is taken to be a conclusive diagnosis for leishmaniasis [35]. However, depending on the skill of technicians, it has been reported that the sensitivity of microscopic examination is between only 42% and 70% [36,37]. Furthermore, such examinations have limitations, in that *Leishmania* species are microscopically indistinguishable. Such observations may also yield false positive results, as artifacts can sometimes erroneously be identified as amastigotes [38]. Consequently, it is not always possible to make a reliable diagnosis. As alternatives, a range of molecular methods have been developed for the diagnosis of leishmaniasis [39,40]. Numerous PCR-based analyses have been described, which are more sensitive than parasite culture, immunohistochemistry, and microscopic examination. PCR provides a sensitive tool for the diagnosis of leishmaniasis and the identification of *Leishmania* species [41], for which the commonly used targets are the mini-exon of nucleus DNA, heatshock protein 70 (hsp70), mini-circle kinetoplast DNA (kDNA), and internal transcribed space (ITS1 and ITS2) ribosomal RNA. In clinical samples, PCR-RFLP for ITS1 and 5.8 S RNA has been demonstrated to be an effective approach for the identification of *Leishmania* species [42]. Several commercial kits based on PCR testing and identification of *Leishmania* species have been developed [39], and, more recently, whole-genome sequencing (WGS) has been used for the diagnosis of travel-related leishmaniasis in an immunosuppressed patient [43]. The advantages of WGS are that it does not require parasite cultivation and can be used to detect variants of parasite genomes in clinical samples [44].

In the Republic of Korea, there have to date been 25 imported cases of leishmaniasis reported, with the most recent case being identified in 2013 [7,8,9,10,11,12,13,14,15]. Of these cases, owing to the technical limitations of PCR, diagnosis was made by confirming the amastigote of *Leishmania* by microscopic examination. Furthermore, unidentified *Leishmania* species have resulted in a prolonged treatment period. In this study, the travel-related acquisition of CL was confirmed based on evidence of the amastigotes in tissues and identification of the species *L. infantum* via PCR amplification and a subsequent BLAST search. Although it was not possible to ascertain the geographical origin of *Leishmania* based on these results alone, given the patient’s travel history to an endemic region and the associated clinical symptoms of *Leishmania*, we were able to determine the suspected country of origin with reasonable confidence. Consequently, when diagnosing leishmaniasis in non-endemic areas, physicians should carefully assess the incubation period of the patient’s disease based on their symptoms and travel history.

As previously mentioned, CL is the most prevalent clinical manifestation of leishmania; however, it is typically a neglected disease, given that it is generally self-healing and rarely fatal [45]. CL is predominantly caused by *L. major*, *L. tropica*, *L. donovani,* and *L. aethiopica* in the Old World. However, *L. mexicana*, *L. amazonensis*, and *L. braziliensis* have been implicated as causal agents of CL in the New World. In contrast, the causative agent of the CL reported in this study was identified as *L. infantum*, which has been established to cause VL. Historically, atypical CL due to *L. infantum* has been reported from France in 1980 [46], and more recently, *L. infantum* has been identified as the causal agent of CL in cases from several regions [4,5,6,47,48]. Given these cases of atypical leishmaniasis, accurate species identification is vital to enable the most appropriate course of treatment. With respect to the case reported herein, on the basis of our understanding of the species-specific information for *Leishmania*, appropriate treatment was administered to the infected patient, as confirmed by the improvement in her condition.

The incubation period for CL is generally known to last from 2 weeks to over several months [2,34], with symptoms typically presenting as a small erythematous papule at the site of a sandfly bite, which gradually increases in size up to 10 mm or more, thereby forming a nodule, at which point it can become ulcerous with a raised border. Such lesions can persist for at least a few months [34]. Occasionally, longer periods of incubation have been reported. For example, twins who had traveled to Tuscany were subsequently diagnosed with leishmaniasis, 7 and 15 months later [49]. Moreover, a case of CL caused by *L. infantum* was found to be associated with an incubation period of 19 years [50]. In addition, there have been report of cutaneous leishmaniasis with a long incubation period of 11 to 16 months in Sicily, Italy [51]. Similarly, in the case reported herein, the patient symptoms developed approximately 2 years after returning to Korea from Spain. Thus, given the potentially long incubation period of leishmaniasis, it is essential for physicians to take into consideration past travel to endemic areas.

Cases of leishmaniasis are currently expanding to countries in which it has previously been unknown, due to a number of factors, including climate change and an increase in the volume of international travel. Surveillance of the emerging leishmaniasis situation is accordingly necessary to determine the extent of the disease in both endemic and non-endemic areas, and to monitor the appearance of vectors. Epidemiological efforts should be complemented by training public health professionals to identify the disease and to become acquainted with the appropriate clinical diagnosis, management, and preventive measures.

## 4. Conclusions

The case reported in this study highlights the potential risks posed to individuals with travel-acquired leishmaniasis. Diagnosing leishmaniasis remains a challenge in non-endemic regions, given the unfamiliarity of physicians with the clinical spectrum and treatment options, as well as the limitation of appropriate diagnostic tools. Consequently, physicians should be mindful with respect to travel history, taking into consideration the destination and disease endemicity when diagnosing leishmaniasis. Furthermore, knowledge of specific *Leishmania* species provides important information regarding the risk of the disease and can guide treatment decisions. Although a diagnosis of leishmaniasis may not always be feasible in non-endemic regions, the risk can be mitigated by raising awareness of this emerging problem among physicians and public health officials.

## Data Availability

The data presented in this study are openly available in the Nucleotide database of the National Center for Biotechnology Information (NCBI), GenBank accession number MZ596307.1.
